# A qualitative analysis of cancer patients’ views on facilitators and barriers for the implementation of oncological exercise therapy

**DOI:** 10.1007/s00520-026-10957-8

**Published:** 2026-07-14

**Authors:** Dominik Morlok, Jana Rüter, Mona Illmann, Laura Bieniosek, Christine Welker, Melanie Reitz, Janina Meuer, Thomas Okon, Hansjörg Baurecht, Wolfgang Herr, Tobias Pukrop, Mirko Brandes, Anika Berling-Ernst, Louisa Sell, Wiebke Jensen, Rebecca Zimmer, Heike Schmidt, Florian Wagemann, Renee Stark, Annalena Kleinhenz, Bernardine Madl, Hajo Zeeb, Michael Leitzmann, Freerk T. Baumann, Anne Herrmann

**Affiliations:** 1 Department of Epidemiology and Preventive Medicine, Medical Sociology, University of Regensburg, Regensburg, Germany; 2 Department of Epidemiology and Preventive Medicine, University of Regensburg, University Hospital Regensburg, Regensburg, Germany; 3 Department I of Internal Medicine, Center of Integrated Oncology Aachen Bonn Cologne Duesseldorf, University Hospital of Cologne, Cologne, Germany; 4 Leibniz-Institute for Prevention Research and Epidemiology – BIPS, Bremen, Germany; 5 Department of Oncology, Hematology, BMT With Section Pneumology, Hubertus Wald Tumor Center - University Cancer Center Hamburg, University Medical Center Hamburg-Eppendorf, Hamburg, Germany; 6 Department of Pediatric Hematology/Oncology, Pediatrics III, West German Cancer Center, University Hospital Essen, Essen, Germany; 7 Department for Radiation Medicine, Medical Faculty of Martin Luther University Halle-Wittenberg, Halle (Saale), Germany; 8 University Cancer Center Schleswig-Holstein, University Hospital Schleswig-Holstein, Kiel, Germany; 9 Professorship of Public Health and Prevention, School of Medicine and Health, Technical University of Munich, Munich, Germany; 10 Department of Medicine III and Comprehensive Cancer Center, (CCC Munich LMU), LMU University Hospital Munich, Munich, Germany; 11 School of Medicine and Health, Department for Preventive Sports Medicine and Sports Cardiology, Technical University of Munich, TUM University Hospital, Munich, Germany; 12 Health Sciences Bremen, University of Bremen, Bremen, Germany; 13 Department of Internal Medicine III, University Hospital Regensburg, Regensburg, Germany; 14 Bavarian Center for Cancer Research (BZKF), Regensburg, Munich, Germany; 15 Division of Physical Activity, Cancer Prevention and Survivorship, German Cancer Research Center (DKFZ), Heidelberg, Germany; 16 Medical Faculty, Heidelberg University, Heidelberg, Germany; 17 Medical Faculty Institute of Social Medicine and Health Systems Research (ISMHSR), University Hospital Magdeburg, Magdeburg, Germany; 18 Center for Translational Oncology, University Hospital Regensburg, Regensburg, Germany

**Keywords:** Cancer, Tertiary prevention, Exercise, Physical activity, Implementation

## Abstract

**Purpose:**

Oncological exercise therapy (OET) can enhance physical function and psychosocial well-being of cancer patients. Despite this, OET is still not routinely implemented in cancer care. To address this gap, our study analyzes cancer patients’ perspectives on facilitators and barriers for OET participation.

**Methods:**

This qualitative study employed semi-structured focus groups and was conducted within the IMPLEMENT research consortium. Focus groups were audio-recorded, transcribed verbatim, and analyzed using framework analysis.

**Results:**

Thirty cancer patients (mean age 57 ± 7 years; 53% women) participated, and most were diagnosed with breast cancer (20%), lymphoma or leukemia (20%), or pancreatic cancer (13%). Participants reported being motivated to become physically active, even when no OET was available. However, they received limited information about physical activity (PA), specifically about OET. They also felt there was a lack of OET offers from healthcare providers. Participation could be increased by improved accessibility involving informational and structural factors, fostering adherence by higher perceived safety and self-efficacy, and by providing individually tailored and low-threshold information for patients.

**Conclusion:**

OET could be a valuable opportunity to support patients throughout their cancer trajectory. To increase participation and promote PA uptake, individually tailored support should be provided in terms of personalized guidance by healthcare practitioners and easily accessible OET offers.

**Trial registration:**

German Clinical Trial Register (Deutsches Register Klinischer Studien—DRKS 00032292), registered 19/07/2023. Clinical Trials Registry (NCT06496711), registered 22/05/2024. Bavarian Cancer Research Center (Bayerisches Krebsforschungszentrum (BZKF) DZ-2024–2165-9), registered 07/06/2024.

**Supplementary Information:**

The online version contains supplementary material available at 10.1007/s00520-026-10957-8.

## Introduction

Oncological treatment is often associated with adverse side effects such as fatigue or pain, which can discourage physical activity (PA) among cancer patients [1, 2]. Oncological exercise therapy (OET) holds potential to help maintain PA levels and to support the uptake of exercise among sedentary patients. Briefly, OET is a structured cancer-specific exercise program delivered across all treatment phases and supervised by qualified therapists, either in one-to-one settings or in small groups. The detailed concept of OET is described in the IMPLEMENT protocol [3]. When tailored to cancer patient’s needs, symptoms, cancer type, and treatment-related side effects, OET can improve multiple outcomes throughout the disease trajectory [4–6]. Compared with usual care, it enhances cardiorespiratory fitness, muscular strength, and physical functioning [5, 7] while also reducing cancer-related depressive symptoms, fatigue, and improving quality of life [7, 8]. Beyond OET, regular PA alone reduces treatment-related complications and probability of relapse, and improves treatment tolerance and prognosis [9, 10]. Results of a recent RCT suggest higher survival rates for patients with colon cancer participating in an OET when compared to inactive controls [11]. Despite this, implementation of OET faces various barriers. For instance, Healthcare practitioners’ (HCPs) often view cancer patients as being reluctant to become physically active [12, 13], although patients themselves appear to be ready for PA [14]. But many seem unable to exercise because they felt uninformed [14–16] or ceased to be active due to unwellness after cancer treatment [13].

Since OET is not yet integrated in standard cancer care in Germany [17], a study found that only 30% of German cancer patients participated in OET during treatment [18]. This may be due to a lack of facilities for providing OET, as well as insufficient knowledge among HCPs about exercising with cancer [12, 19], leading to unspecific PA recommendations [17]. The IMPLEMENT protocol provides more details on (referral) structure and current state of OET in Germany [3]. German studies investigating accessibility of OET concluded that more detailed views of cancer patients are needed to fully understand all multifaceted facilitators and barriers affecting participation in OET [16, 20]. To close this research gap, the present study aims to qualitatively explore patients’ perspectives on facilitators and barriers to OET regardless of type of cancer diagnosis. It is part of the baseline assessment of the IMPLEMENT project, a research alliance of eight German university hospitals and universities, financed by the German Cancer Aid. Initiated in 2023, its goal is to increase the number of OET participants while contributing to a more comprehensive care landscape by designing model research sites implementing various strategies that yield improved structures for OET [3]. Study outcomes will guide the development of OET implementation strategies to optimize cancer care.

## Methods

### Study design

In this qualitative study, a convenience sample of cancer patients was recruited in collaboration with eight research institutions (Bremen, Cologne, Essen, Halle (Saale), Hamburg, Kiel, Munich, Regensburg) located in six different federal states in Germany. Semi-structured focus groups were conducted online from February 2024 to August 2024. Focus groups promote interaction and synergy, enabling participants to clarify their views and generate insights that may not emerge in individual interviews [21]. A more detailed description of the methodology is provided in the published IMPLEMENT study protocol [3]. This study followed the COREQ guidelines [22].

### Participants and recruitment

Patients were eligible if they i) were aged 40 years or older and ii) had a current or prior diagnosis of any type and stage of cancer and were receiving active oncological treatment or follow-up care, to capture experiences across the entire cancer trajectory. Separate focus groups were conducted to explore the needs and challenges of young cancer patients (18–39 years). Findings from this cohort will be reported in a separate publication. Cancer patients were recruited via three pathways: direct personal contact by the research team when permissible with employment status, indirect contact through HCPs at IMPLEMENT sites, and outreach via patient representative pools. Patients willing to participate were included regardless of prior OET attendance. All participants received study information and provided written informed consent. Reasons for non-participation included no response, lack of time, and technical issues. No incentives were provided, but patients will receive brief feedback on the findings after completion of the IMPLEMENT project. Ethical approval was initially granted by the University of Bremen, Germany (approval ref. 2023–16) and subsequently approved by the Ethics Committees of the participating institutions.

### Data collection

A semi-structured focus group guide was developed based on a literature search and discussions within an interdisciplinary research team, aligning with the five domains of the RE-AIM model (Reach, Efficacy, Adoption, Implementation, Maintenance) and adapted from qualitative research questions provided on the RE-AIM website [23]. The comprehensibility and validity of the guide were pre-tested with two cancer patients. Slight adjustments were made afterward to reduce linguistic complexity. Two researchers (DM, JR) trained in medical sociology and public health moderated focus groups of about five participants to enable in-depth exploration and sufficient time for each participant to express themselves [24]. Participants were recruited across all study sites to maximize data diversity [25]. Using open-ended questions and prompts, cancer patients shared perspectives and experiences on facilitators and barriers to participation in OET and suggestions to improve its accessibility. Field notes were taken to complement the data. Focus groups were conducted online using ZoomX, and audio-recorded by the software’s built-in recorder. An external institute performed transcription into text files that were pseudonymized for analysis. Audio recordings were deleted after verification of the accuracy of the transcripts.

### Data analysis

Framework analysis according to Gale et al. [26] was used to analyze data. Two researchers (DM, MI) independently analyzed three transcripts using the QDA software MAXQDA 24 [27]. Both coders developed an initial set of codes that were then compared and harmonized among the research team. Codes were grouped into broader categories, which allowed for the identification of overarching themes through an iterative process of comparison and refinement. DM then continued coding using the developed analytic framework. The coded participants’ responses were subsequently discussed among the research team and organized into a framework matrix to identify recurring themes. Theoretical saturation was assumed after a maximum of three focus groups per IMPLEMENT site, as topics became largely repetitive and comparable. Accordingly, data collection was concluded [25].

## Results

### Sample characteristics

30 cancer patients (mean age: 57 years, SD 7, 53% women) participated in 12 focus groups, each consisting of 2 to 5 participants, and lasting an average of 51 min (SD 10.23). Among patients who provided informed consent, 58% ultimately participated in the focus groups. The total number of patients approached could not be determined due to the multi-pathway recruitment strategy. Breast cancer and lymphoma/leukemia were the most frequent diagnoses (20% respectively), followed by pancreatic cancer (13%). 70% of participants reported taking part in OET. Table 1 provides characteristics of participants and research sites involved.

**Table 1:** Sociodemographic characteristics of participants and their recruitment sites

Characteristics	Participants *n* = 30
*Mean age in years (SD)*	57 (7.3)
*Gender (%)*	
Female	16 (53.3%)
Male	14 (46.7%)
*Cancer type (%)*	
Lymphoma/leukemia	6 (20%)
Breast	6 (20%)
Pancreatic	4 (13.3%)
Colorectal	3 (10%)
Esophagus	3 (10%)
Other	8 (26.7%)
*Participating in OET (%)*	
Yes	21 (70%)
No	9 (30%)
*Research site (%)*	
Cologne	8 (26.7%)
Munich (two research sites)	7 (23.3%)
Essen	4 (13.3%)
Regensburg	4 (13.3%)
Hamburg	3 (10%)
Halle	2 (6.7%)
Kiel	2 (6.7%)

*OET *Oncological exercise therapy

Three key themes emerged inductively as higher-order constructs that subsumed most of the topics raised: Accessibility involving informational and structural factors, adherence increased by perceived safety and self-efficacy, and the need for individually tailored and low-threshold information for cancer patients (Tab2). Themes are described in detail below.

**Table 2:** Identified key themes and subthemes

Key themes	Subthemes	
Informational and structural accessibility	*Informational* • Routine information by HCPs • Direct referral pathways • Information sources • Social network	*Structural* • Availability of OET • Distance to OET facilities • Flexible OET formats • Costs
OET as a safe space encouraging adherence	• Qualified therapeutic personnel • Guidance and empowerment • Psychosocial benefits	
Individually tailored and low threshold guidance to access and adhere to OET	• Guidance through healthcare system • Counselling and referral system • Individually tailored exercise programs	

*HCPs* Healthcare practitioners, *OET* Oncological exercise therapy

### Improving informational and structural accessibility of OET

Information preferences varied, with some focus group participants preferring to receive all available information on PA and OET, while others reported preferring less. However, all agreed that cancer patients should be routinely informed by their HCPs about how to become or remain physically active and about the availability of OET. In addition, most patients expressed a desire for direct referral pathways into OET, indicating a need for proactive communication about OET. Many participants would have appreciated receiving initial PA information directly from their treating oncologist.

*And back then, the doctor treating me said: ‘You need to be physically fit to get through this properly.’ And that was a huge motivation for me to start with exercise and PA. (man, 63 y, lymphoma or leukemia)*

Despite this, cancer patients’ needs for information and counseling were often unmet, as some participants reported having received no information about OET. Knowledge of cancer-specific and therapy-specific PA recommendations was commonly insufficient, leaving many participants with ‘*[…] no idea about it at all. Yes, (the doctor) never heard of it (*= *OET programs)’ (woman, 51 y, breast cancer)*, as aptly put by a patient*.* Some participants identified themselves as information seekers expressing that *‘If I hadn't asked, I wouldn't have found out about it now’ (man, 49 y, pancreatic cancer)*. They used diverse information sources, such as searching the internet or responding to printed advertisement, e.g., flyers and posters, and navigated themselves through the care system to join OET offers that met their needs. These patients were conscious about their health and often already exercised before diagnosis. This was perceived to be a facilitator to becoming or remaining physically active after their cancer diagnosis, as a patient put it, *‘[…] because I know how good it feels afterwards (*= *after exercising). […] Well, now I must admit, I've done a lot of sport all my life. Well, I think much more than others […]’ (woman, 64 y, pancreatic cancer)*. Social networks supported accessing OET regardless of information preference as patients’ families, friends or other peer groups (such as other cancer patients) often motivated them to join an OET.

Participants highlighted four main barriers related to structural accessibility: Lack of OET offers from HCPs, spatial distance to a certified OET facility, flexibility of exercise formats, and associated costs. The availability of OET was perceived to be inadequate: *‘If we want to convey to patients how important exercise is, there have to be programs available. And we don’t have them. We don’t have any programs’ (woman, 54y, breast cancer)*. This was linked to long distances many cancer patients had to travel to reach the nearest OET facility: *‘[…] I have no chance with local public transport, because then I would be on the road for six hours […]. The only option is the car’ (woman, 51 y, breast cancer)*. Even in larger cities, availability of OET was perceived to be limited by *‘an actually very little number of offers’ (woman, 60 y, breast cancer)*. Flexible opening hours and exercise formats could increase participation rates. Many cancer patients wished for more individual OET, ideally, with homogenous patient groups, e.g., clustered by age or diagnosis. They felt that this could help them to adapt to individual physical and psychosocial limitations arising from cancer therapy, such as pain, fatigue or depressive symptoms. Associated costs further hindered participation, since OET is not yet routinely covered by all health insurances across all centers and facilities. While privately insured participants had less disadvantages related to costs, statutory insured patients received financial help by actively seeking selective contracts with health insurances after depleting financed physical therapy quota.^1^ Further costs were linked to transportation, including parking fees, fuel, or transport tickets, especially in rural areas with limited OET options. Despite this, when patients reported being convinced of the positive effects of OET on their health and well-being, these costs *‘wouldn’t have been a decisive criterion’ (woman, 63 y, colon cancer)* for many participants. However, they could pose significant barriers for participation to cancer patients reporting lower levels of interest and motivation regarding PA.

### OET as a safe space encouraging adherence to PA

Participants appreciated OET facilities which were led by qualified therapists and which provided access to peers. They described these facilities as a safe space, where they felt comfortable, and where their needs and wishes were understood and taken seriously. Despite physical side effects like hair loss, catheters or stoma, they did not feel exposed or insecure as they might have in non-cancer settings. The empathy and expertise of supervising therapists minimized patients’ uncertainties regarding how to become active during and after treatment, in particular their attitude of ‘*we are working on it; we are researching it. We know what we're doing […]’ (woman, 51 y, breast cancer)*, as a participant illustrated. This helped as many struggled to determine whether their health status was sufficient for exercise: *‘[…] what is good for me at this point? I have a big scar on my stomach that I notice every time the weather changes. Unfortunately, I’m really noticing the chemo at the moment. And these are things that the girls (*= *therapists) can deal with, you know?’ (man, 49 y, pancreatic cancer).* Training schedules tailored to individual needs of cancer patients empowered participants to trust their own bodies to become physically active again and experience accomplishments which increased their enjoyment for exercising and perceived self-efficacy.

*I was barely able to walk when I began there [OET]. And after three weeks, I was so fit that I said: ‘Yes, it has helped me. I want to keep doing this.’ (man, 49 y, pancreatic cancer)*

*I thought it was great. So, I was on fire. [...] all in all, it was a really great package for me, I have to admit. (woman, 57 y, pancreatic cancer)*

Psychosocial benefits also arose from OET’s social network. Meeting like-minded patients, motivating each other and sharing gratitude for participation encouraged them to maintain an exercise routine, regardless of perceived performance. Despite phases of intensive cancer therapy, they felt proud to at least show up for OET and appreciated that *‘Then, of course, you talk to each other and build each other up. I think that’s what many people also find good, right?’ (woman, 53 y, breast cancer)*. However, some patients preferred general PA offers. They argued that cancer-specific exercise therapy put a constant focus on their disease, so they preferred general rehabilitation facilities over OET to feel ‘normal’ again *‘without creating this parallel universe that it’s all about cancer […]’ (woman, 50 y, soft-tissue sarcoma),* as a woman clarified.

### Individually tailored and low threshold guidance to access and adhere to OET

Participants wished for a healthcare system that would better guide them through their treatment and follow-up care, including more proactive and individually tailored counseling on OET and PA. They pictured automated communication pathways between HCPs, cancer support or social services, OET facilities, and health insurance, stating that *‘[…] the whole thing needs to be integrated so that the patient is guided through the process from start to finish and is also checked regularly’ (man, 63 y, lymphoma/leukemia).* Many cancer patients suggested care pathways that include in-person education on PA and OET from the time of diagnosis, highlighting potential benefits and providing information on how to join OET through contact points or local facilities. Participants appreciated active guidance by their treatment team with becoming physically active and highlighted that *‘[…] if it means you'll be in the gym from next week on, then that's what I would have done’ (woman, 50 y, leukemia)*. Qualified therapists could then develop individually tailored exercise programs that are compatible with patients’ health status, psychosocial needs, PA interests and daily routine. Participants also noted the importance of reducing bureaucracy, for instance by enabling automatic reimbursement or confirmation of coverage by health insurance providers. Ideally, OET offers should be free of charge for patients.

*And pleasantly, they handled the whole process with the health insurance company. So that was really great, well you have enough appointments and enough on your plate, you have to find your way with the situation and that was really helpful. (woman, 51 y, breast cancer)*

## Discussion

This study analyzed cancer patients’ views on facilitators and barriers to participating in OET, addressing the need identified in prior German studies for more in-depth patient perspectives on the multifaceted factors affecting participation [16, 20]. Results suggest that information-seeking behavior for OET differed among participants, but more proactive information about exercising was perceived to significantly support the implementation of OET. Existing structural barriers such as lack of certified facilities, spatial distance to facilities, inflexible exercise formats, and associated costs decreased participation in OET. Overcoming these challenges was easier when patients were interested in becoming active and drawing on diverse information pathways. OET was perceived to be a safe space to re-explore PA and increase self-efficacy and confidence. Participants wished for a routine care pathway including repeated low-threshold, individually tailored guidance and access to OET.

### Cancer patients need individually tailored guidance to access OET

The lack of information on PA and OET is in line with previous studies in this field. A longitudinal study found that 70% of cancer patients were not or only generally informed on PA during their disease [15], and 80% lacked a trusted contact to communicate treatment options of complementary therapy use, including OET offers [19]. Fügemann et al. [29] discussed the navigation through the healthcare system for stroke and lung cancer patients and suggested getting information at an ‘office where you can go to’. This illustrates that cancer patients seek a low-threshold, individually tailored and repeated point of contact providing all relevant information that is also near to where they receive therapy. Further studies recommended supporting patients emotionally and administratively, alleviating fear to become physically active and highlighting safety and benefits of exercise for cancer-related outcomes [16, 19, 29]. Individually tailored communication of OET should respect information preferences of cancer patients and initiate motivation to exercise considering physical and psychological conditions. A widespread implementation of communication training for HCPs could help sharpen their sensitivity towards the needs of patients and expand their awareness of the benefits of OET and their counseling skill set. They should also keep in mind to inform frequently and continuously on OET throughout the entire cancer journey since this potentially influences patients’ intention to become active [15, 29]. To support patients throughout the entire course of cancer treatment, individually tailored support could further be provided by specialized link-workers, such as patient navigators [27] or specifically designated PA navigators [30]. Given time constraints and high workloads among HCPs, shifting selected guidance tasks from physicians to link-workers may improve feasibility, although economic implications require further evaluation.

### OET provides a safe space to recover a disrupted embodied self in cancer patients

Apart from guiding cancer patients into OET programs, participants addressed concerns to restore trust in oneself. This is linked to the concept of the embodied self. As a flexible and biologically grounded concept, the self is seen as emerging from the integration of bodily sensations, emotions, and cognitive processes [31]. In cancer patients, this sense of self can be disrupted and represents a decisive factor for whether and how to become physically active. Our study participants stressed that an impaired self-concept reduced their confidence in their bodies and amplified insecurity to exercise. Also, research suggests that treatment-induced changes of physical appearance affect patients’ body image [32]. For instance, they attributed feeling fragile and being out of breath to loss of muscle mass, which, together with pain, made it burdensome to become active [33, 34]. OET may be a safe space holding resources to counteract disruption of participants’ embodied self: Providing a disease-specific environment, participants felt less exposed and stigmatized, while qualified therapists addressed and eased their fears to become active. This enabled them to re-explore their physical capabilities and re-build trust in their bodies. To support this theoretical approach, our assumptions on the association of the embodied self and OET are substantiated by a meta-analysis in breast cancer patients that found an enhanced body image and self-esteem in oncological patients participating in OET compared to inactive controls [35]. Realizing positive changes by PA, motivation to exercise increased and patients felt less sick [36]. These advantages highlight the need to expand qualified facilities and train specialized physical therapists in OET who can address these safety aspects, and support patients in regaining positive body awareness and self-esteem.

### Strengths and limitations

This study gained comprehensive insights into facilitators and barriers of OET as perceived by cancer patients. It involved different cancer entities and a qualitative approach that explored personal views and preferences in depth. Conducting focus groups may have helped to identify new perspectives and insights through interactive discussion among participants [21]. Providing a shared space, it promoted more sensitive and personal disclosures among participants [37]. However, our sample showed some limitations. Focus group sizes were small (2 to 5 participants) due to scheduling difficulties and frequent short-term cancellations (e.g., health or work-related cancellations, missed appointments). Online focus groups may have restricted observation of non-verbal cues and excluded individuals with limited digital literacy. Despite this, our study provides profound data since focus groups in healthcare especially benefit from small groups [21] and data saturation was assured by scaling-up the number of focus groups [37, 37–39]. Selection bias may have occurred as individuals interested in the study’s objective could be more likely to participate [40]. Findings could be limited by underrepresentation of alternative perspectives on OET due to the use of a convenience sample, effects of group dynamics or social desirability in focus groups [4, 41, 42]. To counteract this, moderators used common techniques such as creating a trusting atmosphere, welcoming any opinion, or actively involving quieter participants to group discussions [21, 41, 42]. Lastly, recall bias may be present as some experiences lay in the past. Transferability to other healthcare systems may be limited by the specific features of the German healthcare system, although some internationally observed facilitators and barriers are comparable [12, 43].

## Conclusion

Study participants reported interest in becoming physically active during and after cancer treatment but reported a lack of information on OET and PA. Creating easily accessible, locally available OET programs that are perceived as safe spaces for cancer patients could foster the uptake of PA. There is a need to implement individually tailored, low-threshold guidance on becoming physically active, delivered continuously by HCPs trained in communication and counseling skills to support optimal patient-centered cancer care.

## Supplementary Information

Below is the link to the electronic supplementary material.

ESM 1 (176 KB PD)

## Data Availability

Qualitative data are available from the corresponding author upon reasonable request.

## Footnotes

1 German health coverage is administered via statutory health insurance (SHI) or substitutive private health insurance (PHI); privately insured people benefit from equal or better health services compared to those receiving statutory coverage [28]. For instance, PHI usually covers more physiotherapy sessions if needed or recognizes other treatment options more easily than SHI. Cancer patients with SHI can accept out-of-pocket payments or seek selective contracts with their insurance to cover expenses beyond standard service, e.g. physiotherapy sessions exceeding the standard rate.
