# Supplementary material for: A qualitative analysis of cancer patients’ views on facilitators and barriers for the implementation of oncological exercise therapy
Source: Support Care Cancer. 2026 Jul 14;34(8):762. doi: 10.1007/s00520-026-10957-8 (PMC13368900; doi:10.1007/s00520-026-10957-8)
Supplement: Supplementary file 1 — (176 KB PD) [file 520_2026_10957_MOESM1_ESM.pdf]

Focus group guide

Thank you for participating in this focus group. My name is [X], and I am a researcher in the IMPLEMENT project. We aim to understand why patients participate in oncological exercise therapy (OET) or why they do not. Furthermore, we want to explore factors that support the successful implementation of OET in different facilities.

To this end, we have prepared a set of questions to learn about your personal experiences and opinions. There are no right or wrong answers. Please feel free to share any additional aspects that come to mind.

With your permission, we will audio-record this focus group and take notes to ensure that we don't miss any important points. You are free to skip any question you do not wish to answer. The recording will be transcribed and pseudonymized for analysis to prevent identification of any individuals or institutions mentioned.

1. *Facilitators and barriers*
   - Who are you, and do you take part in an OET for cancer patients? Which type of OET?
   - If you do, what makes it easier for you to participate? What makes it difficult?
     - How do you feel physically since you participate in an OET? To what extent are you capable of being physically active?
     - How do you feel mentally since you participate in an OET? Does this affect your level of physical activity or how much you exercise?
     - What roles do people in your environment play (e.g. spouse, friends)?
     - Do you have (enough) time to take part in an OET?
     - What role do costs of OET play (e.g. co-payment)?
   - If you do not take part in an OET, why do you not participate?
   - How effective do you think OET is for cancer patients?
     - How does it influence disease progression?

2. *Information process*
   - How did you learn about OET for cancer patients?
   - How were you informed about exercise and physical activity in cancer? By whom? When?
   - Were you sufficiently informed about benefits of OET for cancer patients?
   - Who issued the referral? How was it issued?
   - What worked well and what did not?
   - If you participate(d) in OET, what was particularly helpful regarding your participation?
   - Were you generally informed on physical activity in daily life?

3. *Potential for improvement*
   - What needs to be changed so that more cancer patients can take part in OET?
     - Are the training hours adequate?
     - Are enough spots available?
     - Is the location accessible by public transport (e.g. poor connections, long commuting distances)?
     - Is the location accessible by car (e.g. parking availability, long commuting distances)?
     - Is staff support adequate?

- Are staff members well-trained in OET?
- Are equipment and premises adequate?
- Are digital OET options available? What do you think about digital offers?
- Are the costs of OET appropriate?

4. *Change in physical activity behavior*
   - o Which effects did you notice after participating in OET?
     - To what extent did your physical wellbeing improve or not?
     - To what extent did your mental wellbeing improve or not?
   - o Compared to the time before your cancer diagnosis, do you engage in more physical activity or less?
   - o How great is your need to be physically active? Why is that?

5. *Daily life physical activity*
   - o What helps you to be active in your daily life (e.g. going for a walk)? What hinders you?

6. *Outlook*
   - o Thinking about the future, do you plan on being physically active or to change your physical activity behavior? Why? Why not?

Do you have any other aspects on your mind? Anything we did not talk about?

Thank you!
